# Impact of an early, graduated mobilization program on recovery and postoperative outcomes after cardiac revascularization with extracorporeal circulation

**DOI:** 10.3389/fcvm.2026.1801971

**Published:** 2026-07-15

**Authors:** Jiaqi Liu, Mi Jiang, Xuewen Zhao, Yingdan Liang, Ying Liu, Ruijin Pan

**Affiliations:** Cardiac Surgery Department, The First Hospital of Jilin University, Changchun, Jilin Province, China

**Keywords:** cardiopulmonary support, coronary bypass, functional recovery, hospital stay, postoperative complications, progressive rehabilitation

## Abstract

**Objective:**

This randomized controlled trial evaluated the effects of an early, graduated mobilization program on functional recovery (primary endpoint: postoperative day-5 forced vital capacity [FVC]; secondary endpoints: activities of daily living [ADL] scores, handgrip strength, Short Physical Performance Battery [SPPB] scores, postoperative pulmonary complications [PPCs], and postoperative length of stay) in patients undergoing on-pump coronary artery bypass grafting (CABG).

**Methods:**

Between March to September 2025, 92 patients underwent on-pump CABG; after screening, 80 consecutive eligible patients (sample size calculated based on PPC incidence, *n* = 42 per group before attrition adjustment) were randomized 1:1 using a random number table with allocation concealment to a control group (*n* = 40) or an intervention group (*n* = 40).

**Results:**

On postoperative day 5, the intervention group demonstrated significantly better FVC, ADL scores, handgrip strength, and SPPB scores (all *P* < 0.05). PPC incidence was markedly lower (10% vs. 62.5%, *P* < 0.0001), and postoperative length of stay was shorter (7.13 ± 2.36 vs. 9.18 ± 2.66 days, *P* = 0.0006).

**Conclusion:**

The early graduated mobilization program was safe, feasible, and associated with improved short-term functional recovery, reduced PPCs, and shorter postoperative hospital stay after on-pump CABG. Larger multicenter studies with longer follow-up are required to confirm long-term safety and effectiveness.

## 1 Introduction

In 2024, China conducted 360,765 cardiothoracic and vascular surgeries across 789 hospitals, with 201,257 under cardiopulmonary bypass (CPB), accounting for 55.8%. Coronary artery bypass grafting (CABG) totaled 80,016 cases, an 11.1% increase (8,008 cases) from 2023, representing 22.2% of total surgeries (1.1% increase in proportion) (1). CABG remains the main treatment option for patients with multivessel coronary disease because it restores myocardial perfusion and improves survival (2). Post-CABG cardiac rehabilitation is Class A evidence and Level I recommendation in major guidelines (3). However, rehabilitation participation rates post-CABG remain low at 15% (4). Identifying barriers to cardiac rehabilitation post-CABG is crucial. Post-CABG pulmonary complications (PPCs) occur at higher rates than in general surgery, being a major cause of morbidity, mortality, prolonged ICU stays, and increased economic burden (5–7). Use of CPB during CABG allows complete coronary revascularization and is linked to better long-term outcomes than off-pump techniques, but CPB can cause a range of lung injuries, and in severe cases patients may develop acute respiratory distress syndrome or respiratory failure with reported mortality rates of 50% to 70% (8, 9). Studies show that post-CABG complications lead to declines in function, pulmonary capacity, and muscle strength at discharge and 6 months (10). Preoperative poor physical fitness increases postoperative pulmonary complication risks (11). However, studies on early physical exercise interventions for muscle strength, vital capacity, and pulmonary complications post-CABG are limited. This study explores an early physical rehabilitation intervention in CPB-CABG patients, evaluating effects on muscle strength, vital capacity, and pulmonary complications, with satisfactory rehabilitation outcomes.

## 2 Materials and methods

### 2.1 General data

During the study period (March–September 2025), 92 patients underwent on-pump CABG at the First Hospital of Jilin University. After applying eligibility criteria, 80 consecutive patients were enrolled. This study took the incidence of pulmonary complications as the primary outcome indicator, so a sample size estimation method comparing two sample rates was adopted. Referring to statistical standards, taking *α*=0.05 and 1-*β*=0.90, considering that when the total sample size is constant, n1 = n2=½N, the power of hypothesis testing is the greatest. Therefore, the sample size calculation formula is adopted: $n=\frac{\left(Z_{1-\alpha /2}+Z_{1-\beta }{)}^{2}\cdot 2P\;(1-P\right)}{{\left(P_{1}-P_{2}\right)}^{2}}$ where P1 and P2 are sample rates, and *P* is the average rate of the two samples. Based on the data collected by the project team in the early stage that the incidence of grade II and above pulmonary complications in this center was approximately 80%, it is expected to be reduced to 45% after the intervention. The PASS software was used to estimate the required sample size of 35 cases for each of the two groups. Considering that the loss to follow-up rates of the control group and the intervention group were 20% respectively, the sample size required for each group was at least 42 cases. We finally enrolled 40 patients per group after balancing feasibility and statistical power. The allocation sequence was generated by an independent statistician not involved in the study. Sealed opaque envelopes were used to ensure allocation concealment. A dedicated research nurse performed enrollment and group assignment.

Inclusion criteria were: (1) Age ≥ 18 years; (2) undergoing CABG by median sternotomy with CPB; (3) providing written informed consent and agreeing to functional assessments and training. Exclusion criteria were: (1) concomitant valve surgery; (2) prior chronic obstructive pulmonary disease or preoperative postpericardiotomy syndrome; (3) impaired limb mobility or cognitive impairment that prevented completing questionnaires; (4) unstable hemodynamics or any condition requiring urgent resuscitation; (5) incomplete clinical records; and (6) pregnancy or breastfeeding. Baseline demographic and clinical variables did not differ significantly between the two groups (*P* > 0.05), showing group comparability (Tables 1–3). The study was approved by the Hospital Ethics Committee (Approval No. 25K084-001). Written informed consent was obtained from each patient or their legal surrogate.

**Table 1:** Comparison of baseline characteristics between the two groups.

Group	Number of Cases	Gender (*n*) Male/Female	Age (years, x¯ ± s)	Body Mass Index (kg/m^2^, x¯ ± s)
Control	40	12/28	60.2 ± 9.27	25.81 ± 3.46
Intervention	40	13/27	62.9 ± 8.69	25.63 ± 3.12
*P* value		0.809	0.189	0.817

**Table 2:** Comparison of life style between the two groups.

Group	Number of Cases	Smoking [*n* (%)]	Alcohol Consumption [*n* (%)]	Hypertension [*n* (%)]	Diabetes Mellitus [*n* (%)]	Exercise [*n* (%)]
Control	40	17 (42.5)	9 (22.5)	24 (60)	13 (32.5)	13 (32.5)
Intervention	40	15 (37.5)	3 (7.5)	19 (47.5)	15 (37.5)	8 (20)
*P* value		0.648	0.060	0.262	0.639	0.204

**Table 3:** Comparison of clinical and surgical data between the two groups.

Group	Number of Cases	Ejection Fraction (%)	Operative Time (h)	Number of Grafts	CPB Time (h)	Mechanical Ventilation Time (h)	ICU Stay Duration (h)
Control	40	60.38 ± 5.53	4.95 ± 0.64	3.63 ± 0.56	1.83 ± 0.47	7.51 ± 3.43	42.36 ± 18.21
Intervention	40	58.75 ± 7.32	4.73 ± 0.81	3.65 ± 0.53	1.92 ± 0.52	7.15 ± 2.39	35.14 ± 21.82
*P* value		0.272	0.187	0.854	0.455	0.597	0.117

### 2.2 General information questionnaire

A researcher-designed general information questionnaire was used to record patients’ basic perioperative information, including age, gender, body mass index, pulmonary function test parameters, left ventricular ejection fraction, smoking history, alcohol consumption, comorbidities, activities of daily living (ADL, Barthel Index), exercise habit (defined as regular moderate-intensity physical activity ≥3 times per week, ≥30 min per session in the past year), operative time, number of bypass grafts, duration of mechanical ventilation, and postoperative hospital stay.

### 2.3 Control group intervention: pathway-based rehabilitation nursing

The control group received standard pathway-based rehabilitation nursing, which included the following components: (1) Health Education delivered from the day of admission using instructional manuals and short videos available on the department's official social media account. (2) Psychological Guidance using the Hospital Anxiety and Depression Scale (HADS) on admission and before discharge. Trained nurses delivered structured psychological support in 15–20 min sessions, 2–3 times per week throughout the perioperative period. (3) Measurement of relevant parameters including Short Physical Performance Battery (SPPB), handgrip strength, vital capacity, ADL, and HADS. (4) Rehabilitation Training: Family members received preoperative instruction on the active cycle of breathing technique (ACBT), effective expectoration, chest percussion, ankle pump exercises, and peripheral circulation exercises. After surgery, patients were supervised and encouraged to ambulate early.

### 2.4 Intervention group: early physical rehabilitation program

The intervention group received an early physical rehabilitation program, which was implemented as follows:

#### 2.4.1 Rehabilitation team formation

The rehabilitation team was led by the cardiac surgery head nurse and included two attending physicians, two nursing graduate students, two cardiopulmonary rehabilitation specialist nurses (with formal training in cardiopulmonary rehabilitation), and one physical therapist. The head nurse coordinated the team; physicians assessed pulmonary complications; graduate students supported evidence retrieval; specialist nurses delivered interventions and collected data; and the physical therapist provided movement guidance and corrections. Outcome assessors for grip strength, vital capacity, and PPCs were blinded to group allocation through physical separation.

#### 2.4.2 Program development

The databases were searched according to the ‘6S’ evidence pyramid model, from top to bottom. The following databases and resources were searched: UpToDate, BMJ Best Practice, GIN, ISHLT, SIGN, JBI, Medlive, Cochrane Library, PubMed, Embase, Web of Science, CNKI, Wanfang, CBM, CMJD, and VIP, for evidence on postoperative management after CPB-CABG, including guidelines, consensus statements, systematic reviews, evidence summaries, and meta-analyses. The retrieved evidence informed the development of the physical intervention program, which included training on the use and scoring of assessment scales.

#### 2.4.3 Program implementation

The graduated mobilization program (Table 4) was implemented in stages as follows: (1) Upon achieving hemodynamic stability (systolic blood pressure 90–140 mmHg, heart rate 60–100 bpm without high-dose vasopressors), starting 6 h after ICU admission, patients underwent passive limb movements, ankle pumps, and knee/elbow flexions (10 repetitions per set, 2 sets per day). (2) After extubation, for patients with muscle strength <3, active-assisted contractions (knee/elbow flexions, ankle pumps; 10 repetitions per set, 2 sets per day) were performed. (3) After extubation, for patients with muscle strength ≥3, active-assisted contractions and resistance training (straight-leg raises, arm raises; 10 repetitions per set, 2 sets per day) were conducted. (4) From 24 h after extubation, bed sitting (5 min), resistance training (5 min), and respiratory muscle training using ACBT (2 sets per day) were added. (5) On the day of transfer to the ward, the program included bed sitting for 10 min, bedside sitting for 10 min, standing for 5 min, ward walking for 5 min, and respiratory training (using the ACBT; 2 sets per day). (6) On the first day in the ward, the program included bed sitting for 30 min, bedside sitting for 10 min, chair sitting for 10 min, corridor walking for 50–100 meters, resistance training for 10 min (using 0.5 kg dumbbells: standing elbow flexions, arm raises, and alternating knee bends), grip training (using grippers with progressive intensity), and respiratory training (using the ACBT; 2 sets per day). (7) From the second day in the ward onward, the program included bed sitting for 45 min, bedside sitting for 30 min, corridor walking for 100–200 meters, grip training (as above), warm-up exercises for 5 min (head/neck/shoulder/knee mobilizations), and resistance exercises (using seated resistance bands or dumbbells, with one modality chosen; 2 sets per day). Training was interrupted if any of the following occurred: SBP <90 or >160 mmHg, HR <50 or >120 bpm, SpO2 < 92% on supplemental oxygen, new arrhythmia, chest pain, or patient request.

**Table 4:** Graduated early mobilization program for the intervention group.

Stage	Timing	Main Activities	Duration/Repetitions	Progression/Interruption Criteria	Responsible Personnel
1	6 h after ICU admission	Passive limb movements, ankle pumps, knee/elbow flexions	10 reps/set, 2 sets/day	Progression: Hemodynamic stability (SBP 90–140 mmHg, HR 60–100 bpm, no high-dose vasopressors) Interruption: SBP <90 or >160 mmHg, HR <50 or >120 bpm, SpO₂ < 92% on oxygen, new arrhythmia, chest pain, patient refusal	Cardiothoracic specialist nurses+Physical therapist
2	After extubation+muscle strength <3	Active-assisted contractions (knee/elbow flexions, ankle pumps)	10 reps/set, 2 sets/day	Muscle strength <3	Specialist nurses
3	After extubation+muscle strength ≥3	Active-assisted+resistance training (straight-leg raises, arm raises)	10 reps/set, 2 sets/day	Muscle strength ≥3	Specialist nurses
4	24 h after extubation	Bed sitting, resistance training, ACBT respiratory training	Bed sitting 5 min+resistance 5 min+ACBT 2 sets	Stable vital signs	Specialist nurses
5	Day of ward transfer	Bed sitting 10 min, bedside sitting 10 min, standing 5 min, ward walking 5 min, ACBT	As above	Tolerates previous stage	Specialist nurses
6	Ward Day 1	Bed sitting 30 min, bedside sitting 10 min, chair sitting 10 min, corridor walking 50–100 m, resistance training with 0.5 kg dumbbells, grip training, ACBT	10 min resistance+grip training+ACBT 2 sets	Tolerates previous stage	Specialist nurses+Physical therapist
7	Ward Day 2 onward	Bed sitting 45 min, bedside sitting 30 min, corridor walking 100–200 m, warm-up exercises, resistance training (bands or dumbbells), grip training, ACBT	As tolerated	No signs of intolerance	Specialist nurses+Physical therapist

### 2.5 Observation indicators and evaluation criteria

All functional assessments were performed by cardiothoracic rehabilitation specialist nurses who were blinded to group allocation where feasible. Muscle strength was graded using the standard 0–5 Medical Research Council scale. Detailed descriptions of each outcome measure (vital capacity, ADL, handgrip strength, SPPB, and PPCs) are provided below.

#### 2.5.1 Muscle strength grade

Muscle strength was graded and assessed by specialized nurses in patients in both groups. The grading scale was as follows: Grade 0 (complete paralysis), Grade 1 (visible or palpable muscle contraction but no joint movement), Grade 2 (limb movement possible with gravity eliminated, but unable to overcome gravity), Grade 3 (limb movement possible against gravity, but not against resistance), Grade 4 (movement possible against some resistance, but weaker than normal), and Grade 5 (normal muscle strength).

#### 2.5.2 Vital capacity

Vital capacity (forced vital capacity, FVC) was measured by cardiothoracic rehabilitation specialist nurses using a handheld electronic spirometer (Micro Medical, UK). Measurements were performed according to American Thoracic Society guidelines with the best of three acceptable efforts recorded.

#### 2.5.3 ADL scale

The ADL scale was administered by specialized nurses to assess the ADL in patients in both groups. This scale evaluates eight domains: feeding, bathing, grooming, dressing, bowel and bladder control, toileting, bed-to-chair transfer, and ambulation on level surfaces. Scores range from 0 to 100, with higher scores indicating better self-care abilities.

#### 2.5.4 Handgrip force evaluation

Trained nursing staff assessed handgrip strength in participants using a portable digital dynamometer. Measurements were conducted separately for the dominant and non-dominant hands. Participants sat and were asked to apply gradual, consistent pressure to the device handle. Each hand was tested two times, and the mean result was recorded for analysis.

#### 2.5.5 SPPB

Trained nurses performed the Short Physical Performance Battery to assess lower extremity physical function in participants from both groups. This tool, developed by Guralnik *et al*. (12) in 1994, includes three separate subtests that measure balance, walking speed, and lower limb strength. (1) Balance Test: Participants must hold three positions: side-by-side, semi-tandem, and tandem. Holding the side-by-side or semi-tandem position for 10 s gives 1 point for each, but failure or refusal gives 0 points. For the tandem position, holding it for 10 s gives 2 points, 3–9 s gives 1 point, and less than 3 s (or if the participant cannot try) gives 0 points. The total balance score ranges from 0 to 4. A higher score indicates better balance. (2) Gait Speed Test: The time taken to walk 4 meters was recorded. Two trials were performed per patient, and the faster time was used for scoring. Scoring was as follows: ≥0.83 m/s = 4 points; 0.65–0.82 m/s = 3 points; 0.47–0.64 m/s = 2 points; <0.46 m/s = 1 point; unable to complete=0 points. A higher score indicates better walking ability. (3) Chair Stand Test: Participants stood up and sat down five times as fast as possible, with arms folded across the chest. Scoring is based on time: ≤11.19 s gives 4 points; 11.20–13.69 s gives 3 points; 13.70–16.69 s gives 2 points; >16.70 s gives 1 point; and 0 points if participants could not complete five stands or took more than 60 s. The total SPPB score is the sum of the three subtests and ranges from 0 to 12 points.

#### 2.5.6 Assessment of PPCs

One day prior to discharge, a cardiac surgeon evaluated the clinical symptoms and pulmonary computed tomography findings in the patients in both groups. PPCs that occurred during hospital stay were identified using standardized criteria (13), as detailed in Table 4. Cases with only imaging abnormalities and no symptoms or auscultation findings were classified as subclinical (Grade 1). Clinical complications needed at least two features for Grade 2, or one feature that met criteria for Grade 3 or 4 (14).

### 2.6 Data analysis

All statistical analyses were performed using SPSS software, version 26.0. Nominal and ordinal variables were described by counts and percentages. Normally distributed continuous variables were reported as mean ± standard deviation (SD). Non-normally distributed continuous variables were reported as median and interquartile range. Group comparisons used the independent samples t-test for normally distributed data or non-parametric tests if the data were not normal. For repeated-measures data (ADL, grip strength, SPPB), mixed-model repeated-measures ANOVA was used with time, group, and time × group interaction effects. Mauchly's test was applied to assess sphericity; Greenhouse-Geisser correction was used when violated. Missing data were handled by last-observation-carried-forward. Group comparisons used the independent samples t-test. Statistical significance was set at *P* < 0.05.

## 3 Results

### 3.1 Vital capacity

Before surgery, vital capacity was similar in the intervention and control groups (*P* > 0.05). After surgery, however, vital capacity was higher in the intervention group than in the control group (*P* < 0.05). Detailed data are presented in Table 5.

**Table 5:** Operational definitions of PPCs (13).

Item	Content
Grade 1 Pulmonary Complications	(1) Dry cough; (2) Microatelectasis: Abnormal lung findings and a temperature >37.5 ℃ without other documented causes; chest radiograph findings that are normal or not performed; (3) Dyspnea not attributable to other causes.
Grade 2 Pulmonary Complications	(1) Productive cough not attributable to other causes; (2) Bronchospasm: New-onset wheezing or pre-existing wheezing requiring a change in treatment; (3) Hypoxemia: Alveolar-arterial gradient >29 mmHg accompanied by symptoms of dyspnea or wheezing; (4) Atelectasis: Confirmed by radiological imaging plus a temperature >37.5 ℃ or abnormal lung findings; (5) Transient hypercapnia requiring treatment, such as administration of naloxone or increased manual or mechanical ventilation; (6) Adverse reaction to pulmonary medication.
Grade 3 Pulmonary Complications	(1) Pleural effusion requiring thoracentesis; (2) Suspected pneumonia: Radiological evidence without bacteriological confirmation; (3) Pneumonia: Radiological evidence plus pathogenic microorganisms confirmed by Gram stain or culture; (4) Pneumothorax; (5) Postoperative reintubation or tracheal intubation with assisted ventilation for ≤48 h.
Grade 4 Pulmonary Complications	(1) Respiratory failure: Postoperative assisted ventilation for >48 h, or assisted ventilation for >48 h following reintubation.

### 3.2 ADL scores

Before surgery, ADL scores were similar between the intervention and control groups (*P* > 0.05). On the day 5 after surgery, however, ADL scores were higher in the intervention group than in the control group (*P* < 0.05). Detailed data are presented in Table 6.

**Table 6:** Comparison of vital capacity between the two groups (mean ± SD, mL).

Vital Capacity (mL)	Control	Intervention	t-value	*P*-value
Preoperative	2,162 ± 670.28	2,034 ± 563.30	0.916	0.363
Postoperative	1,832 ± 665.94	2,207 ± 403.20	3.002	0.0036

### 3.3 Grip strength in both hands

Before surgery, grip strength in both the dominant and non-dominant hand was similar between the two groups (*P* > 0.05). On postoperative day 5, grip strength in both hands was greater in the intervention group than in the control group (*P* < 0.05). The improvement was greater in the right hand. Detailed data are presented in Tables 7, 8.

**Table 7:** Repeated-measures analysis of variance for ADL scores between the two groups.

Group	*n*	Preoperative	Ward Day 1	Ward Day 2	Ward Day 3	Ward Day 4	Ward Day 5	Discharge Day
Control	40	99.38 ± 1.68	64.25 ± 4.74	73.25 ± 5.83	78 ± 6.96	83.13 ± 6.76	88.25 ± 5.61	90.63 ± 4.11
Intervention	40	99.88 ± 0.79	70.13 ± 4.00	77.13 ± 3.74	82.88 ± 4.22	87.63 ± 3.92	92.38 ± 2.99	93.98 ± 5.72
t-value		−1.71	−5.99	−3.54	−3.79	−3.64	−4.1	−3.01
*P*-value		0.092	<0.001	0.001	<0.001	0.001	<0.001	0.004

F-time = 654.53, P-time < 0.001; F-group = 32.34, P-group < 0.001; F-interaction = 3.85, P-interaction < 0.001.

**Table 8:** Repeated-measures analysis of variance for right-hand grip strength between the two groups (mean ± SD, kg).

Group	*n*	Preoperative	Ward Day 1	Ward Day 2	Ward Day 3	Ward Day 4	Ward Day 5	Discharge Day
Control	40	20 ± 7.19	15.32 ± 5.98	16.62 ± 5.56	17.82 ± 5.40	18.36 ± 4.32	19.23 ± 4.35	19.78 ± 3.93
Intervention	40	19.26 ± 7.38	18.91 ± 6.75	21.12 ± 6.86	20.82 ± 4.94	21.50 ± 6.34	22.92 ± 6.09	22.6 ± 6.25
t-value		−0.46	2.52	3.30	2.59	2.59	3.12	2.42
*P*-value		0.649	0.014	0.002	0.012	0.011	0.003	0.018

F-time = 11.42, P-time < 0.001; F-group = 3.6, P-group = 0.009; F-interaction = 4.25, P-interaction < 0.001.

### 3.4 SPBB scores

Preoperative SPPB total scores were comparable between the two groups (*P* > 0.05). On postoperative day 5, SPPB scores were significantly higher in the intervention group than in the control group (*P* < 0.05). Detailed data are presented in Table 9.

**Table 9:** Repeated-measures analysis of variance for left-hand grip strength between the two groups (mean ± SD, kg).

Group	*n*	Preoperative	Ward Day 1	Ward Day 2	Ward Day 3	Ward Day 4	Ward Day 5	Discharge Day
Control	40	20.82 ± 7.63	14.65 ± 5.44	16.58 ± 6.0	20.8 ± 15.18	18.25 ± 4.8	18.93 ± 4.56	19.7 ± 4.29
Intervention	40	20.24 ± 6.78	18.52 ± 6.11	21.66 ± 11.29	20.26 ± 5.65	20.38 ± 5.7	21.73 ± 5.52	21.08 ± 5.24
t-value		−0.36	3.00	2.51	−0.22	1.81	2.48	1.29
*P*-value		0.718	0.004	0.014	0.830	0.075	0.015	0.202

F-time = 4.55, P-time < 0.001; F-group = 3.6, P-group = 0.062; F-interaction = 2.52, P-interaction = 0.021.

### 3.5 Rates of major pulmonary complications and postoperative hospital stay

The intervention group had a significantly lower incidence of pulmonary complications (Grade II and above) (10% vs. 62.5%, *P* < 0.0001) and a shorter postoperative hospital stay (7.13 ± 2.36 vs. 9.18 ± 2.66 days, *P* = 0.0006) compared with the control group. Detailed data are presented in Tables 10, 11, 12.

**Table 10:** Repeated-measures analysis of variance for SPPB scores between the two groups.

Group	*n*	Preoperative	Ward Day 1	Ward Day 2	Ward Day 3	Ward Day 4	Ward Day 5	Discharge Day
Control	40	**9.85** **±** **2.12**	10.00 ± 2.16	11.18 ± 1.69	11.25 ± 1.82	11.45 ± 1.58	**10.35** **±** **1.73**	10.15 ± 1.76
Intervention	40	**10.25** **±** **1.78**	10.88 ± 1.16	12.10 ± 1.26	12.18 ± 1.41	12.08 ± 1.21	**11.85** **±** **0.92**	11.73 ± 0.60
t-value		−1.92	−2.26	−2.77	−2.54	−2.16	**−4.05**	−4.35
*P*-value		0.059	0.027	0.007	0.013	0.035	**<0.001**	<0.001

F-time = 108.18, P-time < 0.001; F-group = 20.26, P-group < 0.001; F-interaction = 1.55, P-interaction = 0.161.

Bold values indicate statistically significant differences between the two groups (*P* < 0.05).

**Table 11:** Comparison of the incidence of pulmonary complications between the two groups [*n* (%)].

Group	*n*	Pulmonary Complications [*n* (%)]
Control	40	25 (62.5)
Intervention	40	4 (10)
*P*-value		< 0.0001

**Table 12:** Comparison of postoperative hospital stay between the two groups (mean ± SD, days).

Group	*n*	Postoperative Hospital Stay (days)
Control	40	9.175 ± 2.66
Intervention	40	7.125 ± 2.36
*P*-value		0.0006

## 4 Discussion

### 4.1 Progressive physical rehabilitation enhances physical recovery

CPB-assisted CABG, owing to extensive trauma and CPB-induced stress, often results in muscle weakness, reduced endurance, and impaired ADL, severely affecting patients’ postoperative quality of life. The stress-coping model suggests that post-CABG patients with high levels of exercise fear may view cardiac rehabilitation as a high-risk activity, overemphasizing its negative effects and exhibiting rehabilitation barriers (15). Blair *et al*. (16) found that poor awareness of cardiac rehabilitation reduces patients’ participation enthusiasm and adherence. Therefore, healthcare providers should tailor interventions to the needs of diverse post-CABG patients to reduce exercise fear and improve postoperative outcomes (17). The program's design commences with passive movements 6 h after returning to the ICU upon hemodynamic stability to prevent disuse atrophy and promote circulation; following extubation, the intensity is adjusted according to the patient's muscle strength grade (<3: active-assisted; ≥3: active-resistance training via straight-leg/arm raises and grip exercises); upon ward transfer, the program escalates to include sitting/standing/walking exercises with respiratory training, forming a ‘passive-active-resistance-coordination’ progression. The program's mechanism involves the multidimensional regulation of muscle function, metabolism, and coordination, tailored to the patient's tolerance and shifting from passive to active modes to reduce exercise fear and boost adherence. The results showed that the intervention group had higher ADL scores, right- and left-hand grip strength, and SPPB scores on postoperative day 5 (*P* < 0.05). This finding confirms that evidence-based early exercise regulates the timing, intensity, and content of rehabilitation to improve muscle strength, balance, coordination, ADL recovery, and lay a foundation for long-term functional improvement. This finding aligns with the results reported by Martínez-Martínez *et al*. (18), who demonstrated that long-term cardiac exercise enhances patients’ adaptability and movement capacity.

### 4.2 Implementation of the progressive physical rehabilitation training program reduces the incidence of pulmonary complications

According to previous literature, pulmonary complications remain a leading cause of morbidity after cardiac surgery (19). In the present study, the diagnostic criteria for PPCs were comprehensive and included subclinical Grade 1 changes detected on protocol-mandated postoperative CT scans (performed with explicit patient consent). This approach resulted in a higher reported incidence in the control group (62.5%). The intervention group showed a markedly lower incidence (10%, *P* < 0.0001), demonstrating a significant advantage of the early physical rehabilitation exercise program in preventing PPCs. The underlying mechanisms can be primarily attributed to three aspects: first, the direct effects of respiratory muscle training on intervention. Through the ACBT training, the strength of respiratory muscles, such as the diaphragm and intercostal muscles, is enhanced, thereby improving pulmonary ventilation function, promoting sputum expectoration, and reducing the risk of pulmonary infection and atelectasis. Second, the indirect synergistic effects of limb exercise. Early postoperative limb exercise promotes changes in intrathoracic pressure, accelerates the expulsion of residual gas from the lungs, improves pulmonary gas exchange function, and simultaneously enhances systemic blood circulation, reduces pulmonary congestion, and mitigates pulmonary inflammatory responses. Third, the safety assurance provided by the phased training approach. The program adjusts training content based on the patient's postoperative recovery status (e.g., hemodynamic stability and muscle strength grading), avoiding an increased respiratory load caused by premature strenuous exercise and ensuring that the rehabilitation training matches the patient's physical tolerance, thereby fundamentally reducing the probability of pulmonary complications. Furthermore, under the guidance of a professional rehabilitation team throughout the process, patients in the intervention group mastered correct breathing and expectoration techniques, thereby reducing postoperative sputum retention and further lowering the risk of pulmonary infection. This finding is consistent with those of related studies (20), which confirmed that early physical rehabilitation exercise, through the synergistic model of “respiratory muscle training+limb exercise,” can effectively protect pulmonary function, reduce the incidence of PPCs in patients undergoing CABG with CPB, and provide an important guarantee for smooth postoperative recovery.

### 4.3 Implementation of the progressive physical rehabilitation training program shortens postoperative hospital stay

Postoperative LOS is an important indicator of patient recovery efficiency and the efficient utilization of healthcare resources. Patients undergoing CABG with CPB often require a prolonged hospitalization period due to multiple postoperative complications and slow physical recovery, a situation that not only increases the economic burden on patients but also consumes substantial medical resources. The early physical rehabilitation exercise program accelerated patient recovery through multidimensional interventions: on the one hand, rapid physical recovery enabled patients to meet discharge criteria earlier—the restoration of muscle strength and motor ability allowed patients to independently perform basic activities, thereby reducing the burden of postoperative care. On the other hand, the significant reduction in the incidence of pulmonary complications decreased the additional treatment costs and nursing time associated with such complications, thus preventing extended hospitalization due to issues such as pulmonary infection and atelectasis, thereby shortening the hospital stay. The results of this study showed that the postoperative hospital stay in the intervention group was 7.125 ± 2.36 days, significantly shorter than that (9.175 ± 2.66 days) in the control group (*P* = 0.0006), demonstrating that the early physical rehabilitation exercise program effectively shortens postoperative hospital stay and enhances the efficient utilization of healthcare resources. This trend is consistent with the findings of studies (21), in which in-hospital cardiac rehabilitation significantly improved exercise capacity and shortened hospital stay in patients who had undergone cardiac surgery. Shortening the hospital stay not only reduces patients’ hospitalization costs, transportation expenses, and family caregiving costs—thereby alleviating the economic burden on families—but also enables a more rational allocation of limited resources, such as ICU beds, general ward beds, and medical personnel. This approach allows more patients in need of surgical treatment to receive timely medical services, enhances the efficient utilization of medical resources, and promotes multiple benefits, including “patient benefit, hospital efficiency improvement, and societal benefit.”

### 4.4 The progressive physical rehabilitation training program demonstrates safety and clinical application prospects

The early graduated mobilization exercise program demonstrated good safety (no mobilization-related adverse events occurred) and high feasibility (intervention fidelity rate of 92%, defined as sessions completed vs. planned), primarily evident in three aspects: first, the scientific evidence-based basis of the program. The program integrated high-level evidence, such as guidelines, expert consensus statements, and systematic reviews, through searches of multiple authoritative databases encompassing the “6S” evidence pyramid model, ensuring that the training content and timing aligned with the postoperative recovery patterns of patients undergoing CABG with CPB and avoiding unsubstantiated intervention. Second, the professionalism of the team's support. The intervention team, composed of a cardiac surgery head nurse, attending physicians, nursing graduate students, cardiopulmonary rehabilitation specialist nurses, and a physiotherapist, featured a clear division of labor and effective team collaboration. They could assess changes in the patient's condition in real-time, adjust the training plan promptly, and effectively mitigate risks during rehabilitation training. Third, the individualization of the phased training approach. The program dynamically adjusted training intensity and content based on the patient's postoperative muscle strength grading and recovery status, following the principle of “from weak to strong and from passive to active,” ensuring that the training intensity matched the patient's physical tolerance and avoiding exacerbation of the condition due to excessive exercise.

Regarding clinical application prospects, this program holds strong potential for broad dissemination: on the one hand, the program features clear operational procedures and well-defined phased goals, making it easy for medical staff to master and implement, thus rendering it readily applicable to cardiac surgery practices in hospitals at various levels. On the other hand, by targeting common postoperative issues in patients undergoing CABG with CPB, such as physical decline and pulmonary complications, the program establishes an integrated “prevention-intervention-rehabilitation” model. It is not only applicable to the patient population in this study but can also be tailored individually based on the characteristics of different patients (e.g., those with comorbidities such as diabetes or hypertension), thereby expanding its scope of application.

This study has several limitations. First, the sample size was relatively small and it was conducted to a single center, which may limit generalizability. Second, no long-term follow-up was performed after hospital discharge to assess sustained function recovery or readmission rates. Third, although baseline differences in smoking and alcohol consumption were not statistically significant, they could have influenced postoperative outcomes. Fourth, this was an open-label trial with only outcome assessors blinded; performance bias cannot be completely ruled out. Future research should expand the sample size, conduct multi-center studies, further optimize program details to enhance their scientific rigor and applicability, and incorporate follow-up data to evaluate long-term benefits. This would enable the early physical rehabilitation exercise program to better serve patients undergoing CABG with CPB, facilitating their rapid and safe postoperative recovery.

## 5 Conclusion

In conclusion, the early graduated mobilization program was safe and feasible and significantly improved short-term functional recovery, reduced the incidence of postoperative pulmonary complications, and shortened hospital stay in patients undergoing on-pump coronary artery bypass grafting.

## Data Availability

The original contributions presented in the study are included in the article/supplementary material, further inquiries can be directed to the corresponding author.
